# Modeling the Emergence of Circadian Rhythms in a Clock Neuron Network

**DOI:** 10.1371/journal.pone.0033912

**Published:** 2012-03-27

**Authors:** Luis Diambra, Coraci P. Malta

**Affiliations:** 1 Centro Regional de Estudios Genómicos, Universidad Nacional de La Plata, Florencio Varela, Buenos Aires, Argentina; 2 Instituto de Física, Universidade de São Paulo, São Paulo, Brasil; National Research & Technology Council, Argentina

## Abstract

Circadian rhythms in pacemaker cells persist for weeks in constant darkness, while in other types of cells the molecular oscillations that underlie circadian rhythms damp rapidly under the same conditions. Although much progress has been made in understanding the biochemical and cellular basis of circadian rhythms, the mechanisms leading to damped or self-sustained oscillations remain largely unknown. There exist many mathematical models that reproduce the circadian rhythms in the case of a single cell of the *Drosophila* fly. However, not much is known about the mechanisms leading to coherent circadian oscillation in clock neuron networks. In this work we have implemented a model for a network of interacting clock neurons to describe the emergence (or damping) of circadian rhythms in *Drosophila* fly, in the absence of *zeitgebers*. Our model consists of an array of pacemakers that interact through the modulation of some parameters by a network feedback. The individual pacemakers are described by a well-known biochemical model for circadian oscillation, to which we have added degradation of PER protein by light and multiplicative noise. The network feedback is the PER protein level averaged over the whole network. In particular, we have investigated the effect of modulation of the parameters associated with (i) the control of net entrance of PER into the nucleus and (ii) the non-photic degradation of PER. Our results indicate that the modulation of PER entrance into the nucleus allows the synchronization of clock neurons, leading to coherent circadian oscillations under constant dark condition. On the other hand, the modulation of non-photic degradation cannot reset the phases of individual clocks subjected to intrinsic biochemical noise.

## Introduction

Most living organisms present rhythmic phenomena whose periods range from few milliseconds to years. Circadian oscillation is an important example of this kind of phenomenon. Several recent observations have suggested that the circadian rhythm at the molecular level results from a gene regulatory network [1]. In *Drosophila melanogaster* the expression of the circadian clock genes occurs within approximately 150 clock neurons. The molecular mechanisms of these fundamental oscillators consist mainly of two interlocked transcriptional feedback loops involving *per*, *tim*, *clk*, *vri* and *pdp1* genes [2], [3]. In one loop, the CLK protein activates *per* expression, and the PER-TIM complex then inhibits the activity of *clk*. Furthermore, *clk* is regulated negatively by the VRI protein and positively by the protein PDP1. In the second loop, *vri* and *pdp1* are directly activated by the CLK protein. After its synthesis, the PER protein is phosphorylated at several residues. This leads to a time delay between the rise of mRNAs and that of the PER acting as transcriptional repressor for the *clk* gene. Thus alternating protein production, gene repression, and protein degradation may lead to self-sustained oscillations. The circuit is further complicated by the influence of the kinase *doubletime* on degradation and transport of the PER protein [4]. Moreover, the degradation rate of the TIM protein is indirectly controlled by light, which enables entrainment with the environment [5]. Over the past years, a number of deterministic and stochastic models for the circadian clock have been proposed [6]–[11]. They differ largely in the detail of the specific oscillator and, consequently, in their complexity.

Recent observations have revealed a more complex organization in the fly brain. They indicate that the neurons (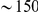) are organized into functional groups. Each group contributes to the control of the rhythmic behavior in an orchestrated manner. In *Drosophila*, the clock neurons have been divided into two major groups: the lateral neurons (LN) and the dorsal neurons (DN). The lateral neurons are subdivided into three subgroups: the large and small ventrolateral neurons (LN
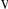
s), and the dorsolateral neurons (LN
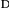
s). There is ample evidence indicating that the small LN
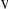
s are especially important as circadian pacemakers for locomotor rhythms in constant darkness (DD) [12], [13]. It is known that the molecular oscillations that underlie circadian rhythms in the small LN
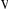
s, persist for weeks in constant darkness [14], [15], while in other *Drosophila* tissues these oscillations damp rapidly in the absence of light [16], [17]. This is an indication that the coherent circadian output results from synchronized electrical activity of the LN group [18], [19] and suggests that the neuronal interactions of the various groups of clock neurons in the fly brain play a critical role in the circadian behavioral rhythms.

Today there is evidence suggesting that the interneuron synchronization in *Drosophila* is achieved through the pigment-dispersing factor (PDF) neuropeptide, which is specifically expressed in the ventral lateral neuron group (LN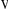) [18], [20]–[22]. Pdf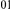 mutant flies gradually lose behavioral rhythms in constant darkness, even though the molecular oscillations persist within individual LN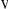 neurons [21], [22]. This loss of oscillatory behavior in pdf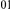 mutant flies has been interpreted as a loss of neuron synchrony due to the noisy nature of the molecular clock machinery. Therefore, in constant darkness, PDF is required to maintain the circadian rhythm of a group of neurons, though it is not required to maintain circadian rhythm in a single neuron [21], [22]. Thus, in the presence of *zeitgebers* the phases of individual clocks are reset, but in their absence, the molecular fluctuations disperse the phase of individual clocks relative to each other, leading to the oscillation damping. Despite this progress towards understanding the genesis of circadian rhythms, the precise action of the synchronizing agent over the clock machinery is essentially unknown at the present time.

The mathematical models mentioned above were developed to explain cell-autonomous oscillations and could not explain some synchronization aspects of the cellular clocks in constant darkness. Models that take into account interactions between the clock cells themselves have been proposed [23]–[28]. More recently, detailed models for a clock neuron network in mammals have also been proposed [29], [30]. These multiscale models provide details of the suprachiasmatic nucleus organization at both the gene regulatory and electrophysiological levels.

In this paper, we present a model of clock network to assess the putative mechanisms through which the clock neurons in the fly brain are coordinated to produce a circadian coherent output even under constant darkness condition. The model consists of an array of connected circadian pacemakers. The individual pacemakers are described by a well-known biochemical model for circadian oscillation [6], to which we have added degradation of PER protein by light and multiplicative noise. Each clock neuron has some of its parameters modulated by the PER protein level of all the other clock neurons. This corresponds to a fully connected network, without self-interactions. In particular we have investigated the effect of modulation of the parameters associated with (i) the control of net entrance of PER into the nucleus; and (ii) the non-photic degradation of PER.

## Methods

### The Model

**Figure 1:**
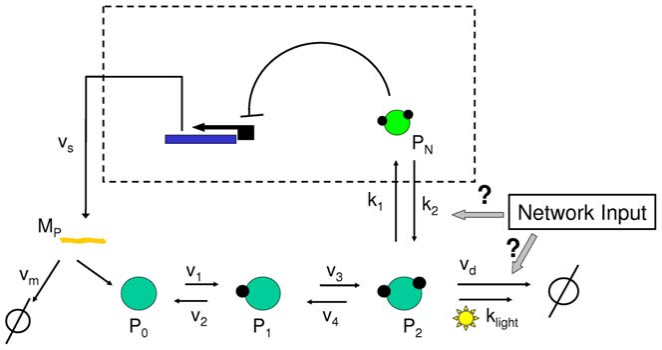
Sketch of the model (adapted from [**7**]). A portion of *per* mRNA (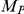) is translated to PER protein in the cytosol (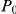), where it undergoes two phosphorylations. Part of the fully phosphorylated PER protein (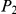) enters into the nucleus (dashed box) and the remains are degraded in the cytosol either induced by light or basally. *Per* activity is inhibited by 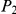 forming a negative feedback loop. The Input Network box represents hypothetical regulation of the 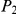 entrance and/or degradation.

To model the synchronization of the oscillations in the small LN
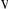
s group, we assume that, at the level of individual clock neurons, the circadian clock is represented by the core negative feedback loop established by PER. For the individual clock neurons we have adapted a model originally proposed by Goldbeter [6] that explicitly includes (i) transcription: the gene is transcribed into mRNA (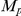) in the absence of phosphorylated PER in the nucleus (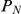), assuming, that the repression is cooperative; (ii) translation: a portion of this mRNA is degraded, and another portion is translated into PER protein (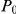) in the cytoplasm; (iii) phosphorylation: PER protein is phosphorylated in a reversible way twice (from 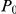 to 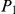 and from 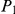 to 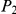); (iv) degradation: the fully phosphorylated PER (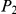) is degraded by the default molecular machinery following a Michaelis-Menten rate expression; (v) transport: the entrance of PER into the nucleus is assumed to be a reversible first-order process. These processes correspond to the model introduced by Goldbeter [6] for autonomous oscillations. In order to simulate the light effect we have added a PER degradation process due to light exposure: the fully phosphorylated PER (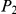) is linearly degraded by light exposure at the rate 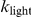. Figure 1 displays the model schematic diagram.

The stochastic biochemical processes underlying the molecular machinery are subject to noise or fluctuations [7], [31]. For this reason, all the above mentioned processes are affected by molecular noise, this noise term being assumed to be multiplicative. The temporal evolution of the above-mentioned chemical species is then governed by the following set of differential equations:
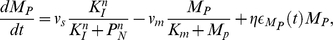


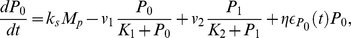


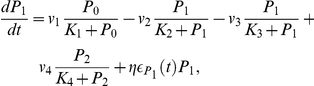


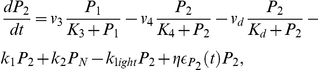


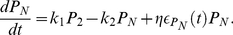
(1)This cellular model incorporates enough details about the PER loop, thus allowing us to test some of the hypotheses regarding the emergence of circadian rhythms in a network of noisy neurons. It should be remarked that the model described by Eqs. (1) is reduced to the original Goldbeter model [6] when 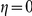 and 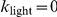. The additional term representing the degradation of PER by light is null during the night. In our simulations this is accomplished by setting the parameter 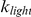 equal to zero. This additional degradation term allows the synchronization of the clocks by an external clue (the light). However, under DD conditions, when there is no light-induced degradation, each clock neuron oscillates almost independently of each other. The molecular fluctuations mentioned above could disperse the rhythmic phase of the clock neurons relative to each other, leading to the loss of synchrony between the oscillator units [7]. In this way the overall oscillatory output of the network is damped.

Neuronal interactions may also be required to maintain proper molecular oscillations in constant darkness. Our model assumes that the clock neurons form a network, and that the interneuron communication between the clock neurons is through the current value of cytoplasmic 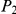 concentration. The input for each clock neuron is the averaged 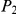 concentration over all other clock neurons with equal weight. So the input signal over each clock neuron is 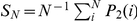, where 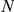 is the number of clock neurons in the network, which was set to be 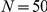 in all cases, unless otherwise specified. This corresponds to a fully interconnected network, without self-interactions. Unlike to the models [23], [25] that use a heterogeneous population of clock neurons, the network of our model is formed by identical cells. In this case, the dispersion in the oscillations' period and phase arises from the molecular fluctuations rather than from a heterogeneous clock population. As the precise mechanism for maintaining circadian regime under free-running condition remains essentially unknown, we will test the following alternative hypotheses:

- The input signal 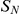 modulates the net rate of the fully phosphorylated PER entrance into the nucleus. This is implemented by increasing and/or decreasing the parameters 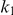 and 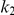: 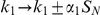, 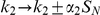.
- The input signal 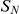 modulates the degradation rate of the fully phosphorylated PER. This is implemented by increasing or decreasing the degradation constant 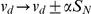.

### Numerical simulations

In our simulations we have considered a network of 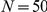 clock neurons, where each clock neuron obeys Eqs. (1). They are connected through the network input that modulates the parameters 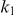, 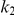 and 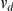, as explained above.

The numerical integration was performed by using the Runge-Kutta method, with the integration time step set to 0.1 min. To obtain fluctuations similar to those exhibited by a stochastic version [7] of the model we have used 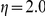. Other parameter values used in all simulations are given in Table 1. The initial condition of the 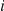-th clock neuron corresponds to the chemical state of a referential clock neuron at the time 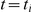, where 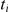 is a Gaussian distributed random number with mean 0 h and standard deviation of 1 h. Thus the initial condition of the network corresponds to an ensemble of pacemakers whose phases are Gaussian distributed around 0 h. Using this initial condition, the network is subjected to 9 days of LD (12 h of light and 12 h of darkness), followed by 7 days under DD condition (24 h of darkness) or free-running condition. In our simulation the absence of light (darkness) is accomplished by setting 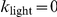.

**Table 1:** Parameter values used in the simulations.

Parameter	value	unit
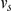	0.50	nM 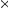 h 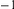
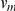	0.35	nM 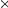 h 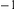
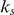	2.00	h 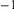
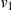	6.00	nM 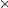 h 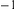
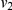	3.00	nM 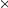 h 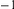
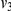	6.00	nM 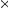 h 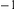
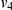	3.00	nM 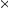 h 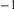
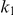	2.00	h 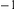
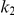	1.00	h 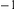
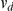	[2.7,3.0]	nM 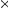 h 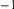
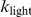	[0.2,8.5]	h 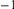
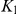	1.50	nM
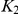	2.00	nM
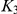	1.50	nM
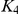	2.00	nM
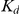	0.10	nM
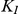	1.50	nM
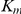	0.20	nM
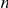	4	
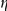	2.0	

Except for 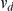, 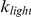 and 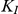, the above values are the same used in [7].

For each set of parameter values we determine the amplitude, the period and the phase of the 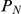 level. This is accomplished by a nonlinear fitting of a cosine function of the form
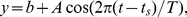
(2)where 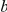 is the baseline, 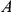 the amplitude, 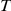 is the period, and 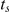 the phase shift. Thus, the phase shift corresponds to the time interval from day onset to the 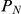 peak. The fitting is done for several fixed values of 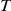 ranging from 16 h to 32 h, with a resolution of 15 min. For each clock neuron, we select the period 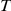 corresponding to the best fitting.

It should be remarked that there are two procedures for obtaining the average period and the average phase of the network: (i) averaging the period and phase obtained for each individual clock neuron over the clock neuron population; (ii) first averaging the 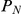 level over the clock neuron population, and then fitting the cosine function for the average level 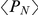. These procedures for obtaining the average phase and period lead to the same results when the clock neurons are well synchronized. However, under DD condition, there is a loss of synchronization that causes a decrease in the amplitude, which that does not allow the precise determination of the average value of the phase and period using the procedure (ii) above. For this reason we have adopted procedure (i) to determine the period and phase of the clock network.

The overall degree of synchronization over a specified time interval is analyzed by computing the parameter 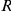 [27]

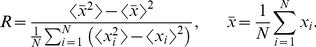
(3)This parameter 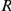 has been computed using the concentration of PER in the nucleus, i.e., 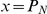. Furthermore, the parameter 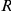, the period 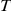, and phase shift 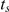 were obtained by averaging over the last 5 days, independently of the case (LD condition or DD condition).

## Results

**Figure 2:**
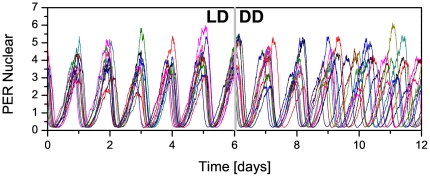
Loss of synchronization for noninteracting clock neurons. Time course of nuclear concentration of PER protein for a group of 10 noninteracting clock neurons for 6 days under LD condition, followed by 6 days under DD condition.

**Figure 3:**
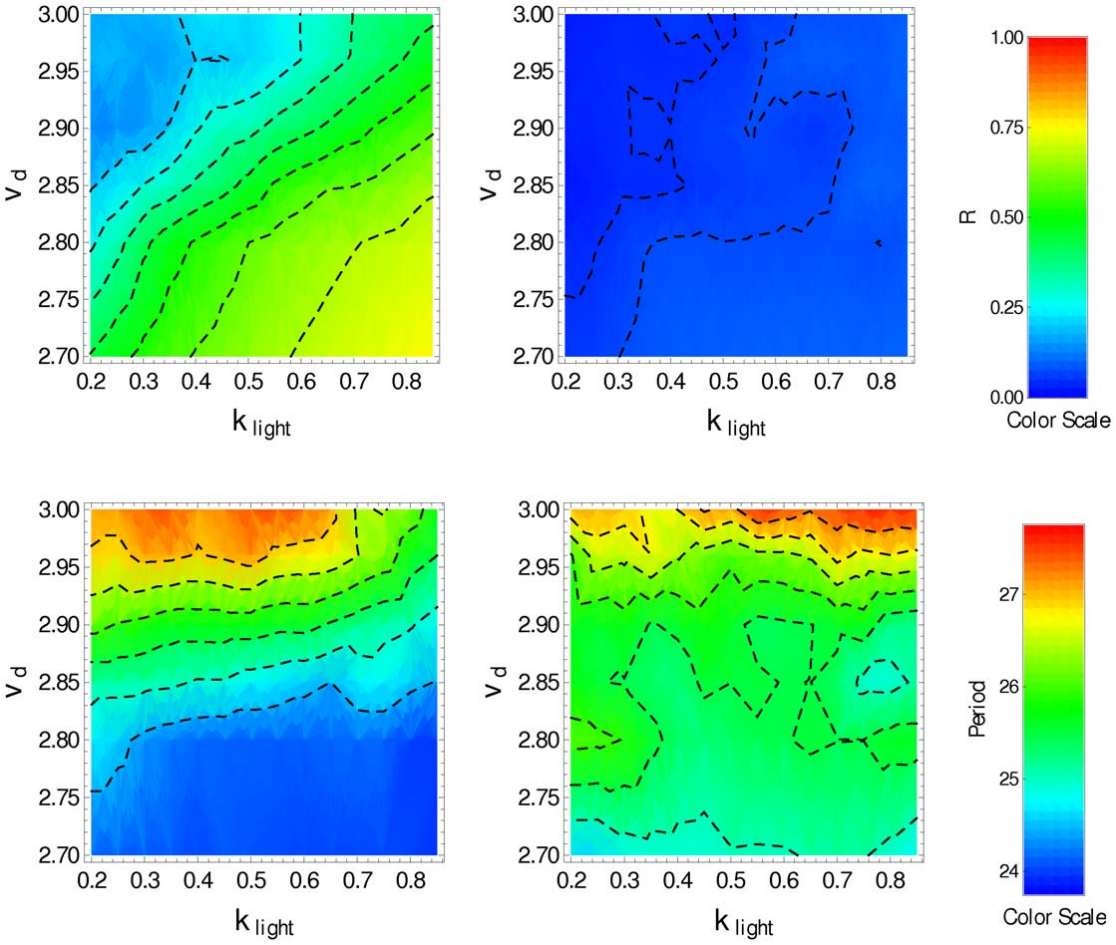
Synchronization degree and period for noninteracting clock neurons. 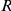
 (top), period (bottom), in the 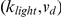-space, obtained by averaging over a set of 50 noninteracting pacemakers in the last 5 days. Plots on the left correspond to LD condition, plots on the right correspond to DD condition (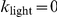).

Figure 2 displays the temporal behavior of 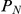 of the noninteracting neuron population for 6 days under LD condition followed by 6 days under DD condition (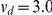 and 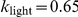, other parameter values being given in Table 1). We can see that in the presence of the *zeitgeber* the clock neurons are synchronized, but in free running (DD condition) there is a loss of synchronization. Nevertheless, the molecular oscillations of each clock neuron are little affected by the absence of the *zeitgeber*. Figure 3 displays, in color scale, the parameter 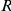 (top row), and the period 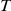 (bottom row) as function of the parameters 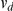 and 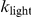 obtained under LD condition (left panels) and under DD condition (right panels). The top-left panel shows that the degradation induced by light allows the synchronization by resetting the phase of individual clock neurons under LD condition. This is evident for high values of 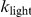 and low values of 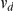 where the parameter 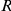 reaches 0.75. In the bottom-left panel (LD condition) we can see that 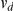 must be smaller than 2.85 to obtain circadian oscillation; for higher values of 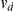 the period is shorter than 24 hs. At the top-right panel one can observe any degree of synchronized behavior, and that oscillations in the range 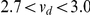 have periods longer than 24 hs.

**Figure 4:**
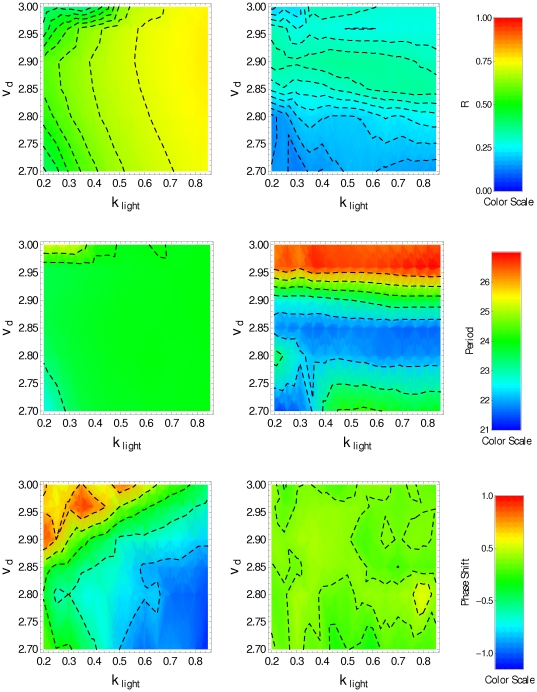
Synchronization degree, period and phase for interacting clock neurons with 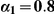
 **.** 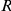 (top), period (center) and phase shift (bottom) in the 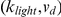-space, averaged over the cell population in the last 5 days when 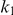 is replaced by 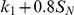. Plots on the left correspond to LD condition, plots on the right correspond to DD condition (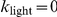).

**Figure 5:**
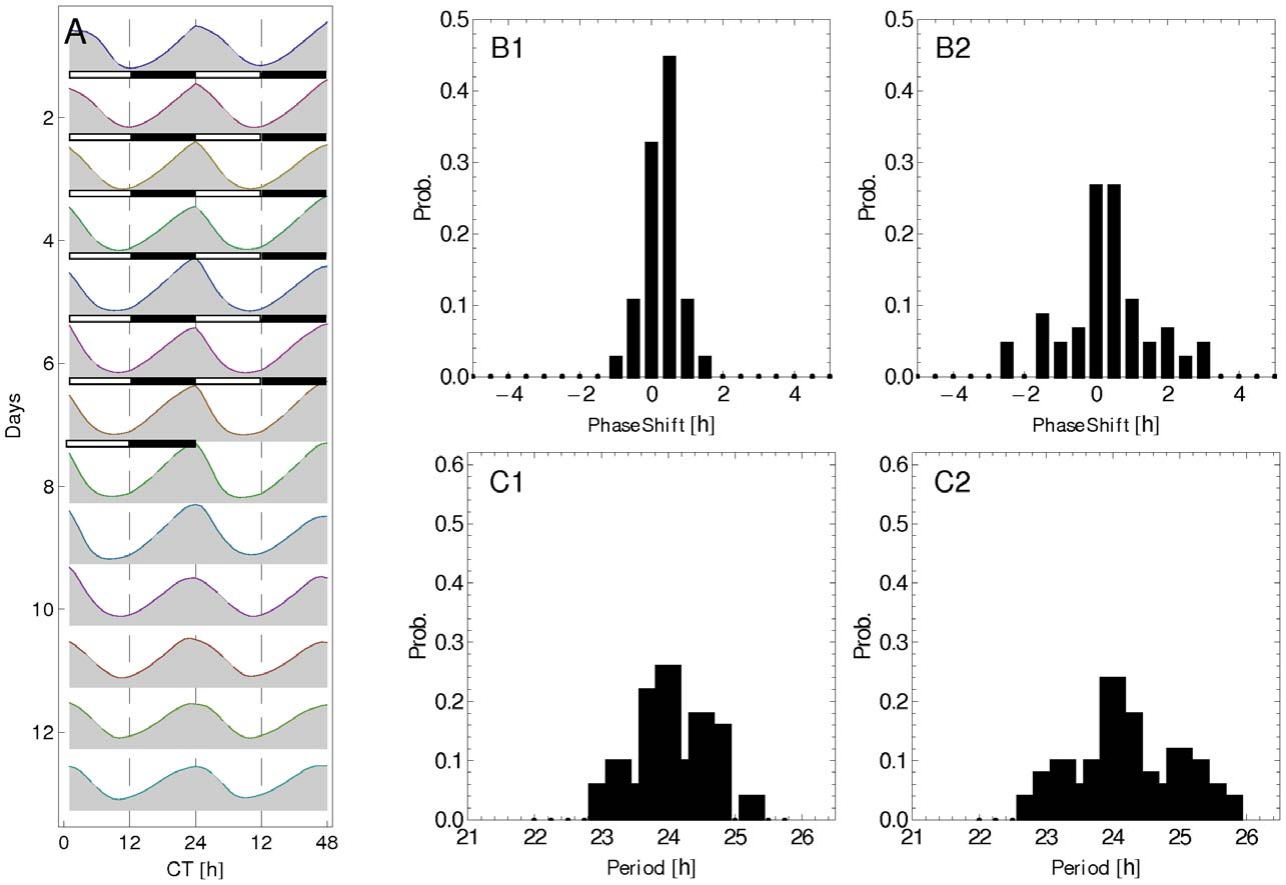
Synchronization for interacting clock neurons: Actogram and histograms. A: Double plot actogram of 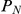 averaged over the neuron population 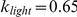, 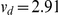, for 7 days under LD condition (indicated by white-black bars) followed by 7 days under DD condition. Histograms of phase shift (B1, B2) and period (C1, C2) obtained by fitting Eq. (2) to the 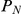 level for each clock neuron. Plots on the left (right) correspond to LD (DD) condition.

Now we will test the hypotheses regarding the effects of interneuron interaction on synchronization:

- We assume that the input signal 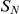 increases the rate of entrance of 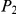 into the nucleus, this being accomplished by replacing 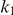 by 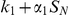 in Eqs. (1). Figure 4 displays, in color scale, the parameter 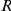 (top panels), the period 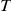 (middle panels) and the phase shift 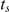 (bottom panels) as function of the parameters 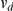 and 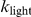, obtained under LD condition (left panels) and under DD condition (right panels) for 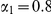. The top-right panel shows that the interneuron interaction allows for some level of synchronization (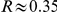) under DD condition in the whole interval of 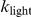 considered in the numerical simulation, with 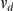 in the interval 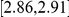. Under LD condition the degree of synchronization is high (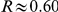) in the whole interval of both parameters 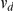 and 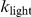. The middle-left panel of Fig. 4 shows that in almost the whole interval considered here, the system presents circadian oscillations under LD condition, while under DD condition (middle-right panel) circadian oscillations, associated with interneuron synchronization, exist only for 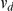 in the interval 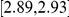 for the whole range of 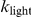. Circadian oscillations are also present in the region corresponding to 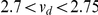, and 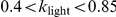. However, in this latter range of parameters, the oscillation amplitude is smaller as a consequence of the low degree of synchronization (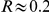), as shown in the top-right panel. Regarding the phase shift 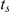, the bottom panels of Fig. 4 show that −1.2 h
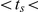
1.0 h under LD condition (bottom-left panel) and −0.5 h
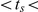
0.5 h under DD condition (bottom-right panel). In order to simulate the behavior observed in *Drosophila*, we look for a region in the 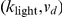-space where the resulting network oscillations have the following features: (i) the 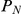 concentration averaged over the clock neuron population has a period close to 24 h, (ii) neuronal synchronization and (iii) the phase difference between the LD and DD conditions is small. Figure 4 shows a small region around 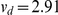 and 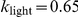 that satisfies these requirements when 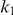 is increased by the interneuron communication. Figure 5A depicts the time course (double plot actogram) of 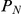 averaged over the clock neuron population for 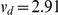 and 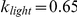. The time course displayed correspond to seven days under LD condition (indicated by the white/black bars) followed by seven days under DD condition. The resulting oscillations preserve the 24 h period under both LD and DD conditions, and there is no phase shift between the LD and DD conditions. A decrease in the oscillation amplitude is observed under the DD condition due to a lower degree of interneuron synchronization.

**Figure 6:**
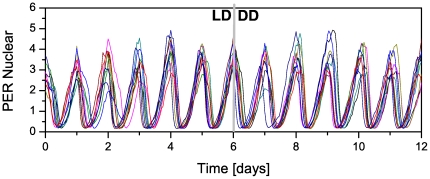
Synchronization for interacting clock neurons. Time course of nuclear concentration of PER protein in 10 interacting neuron clocks (randomly picked from a population of 50) for 6 days under LD condition followed by 6 days in DD condition. 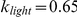 and 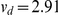, other parameter values are listed in Table 1.

In addition, we have also determined the period and phase of the oscillations of the individual clock neurons by fitting the time course of 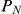 to the function (2). Figure 5B shows the histogram of phase shift (B1 for LD and B2 for DD), and Fig. 5C shows the period histogram of the cell population (C1 for LD condition, and C2 for DD condition). One can observe that our model suggests that the dispersion both in the oscillation period and in the phase shift contributes to a decrease in the amplitude of oscillation of the average 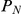. Figure 6 displays the temporal behavior of 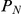 of 10 interacting neurons (from a population of 50 clock neurons) in 6 days during LD condition followed by 6 days during DD condition for 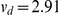 and 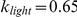. We can see that in free-running (DD) condition the synchronization of the clock neurons is similar to that obtained in the presence of the *zeitgebers*.

**Figure 7:**
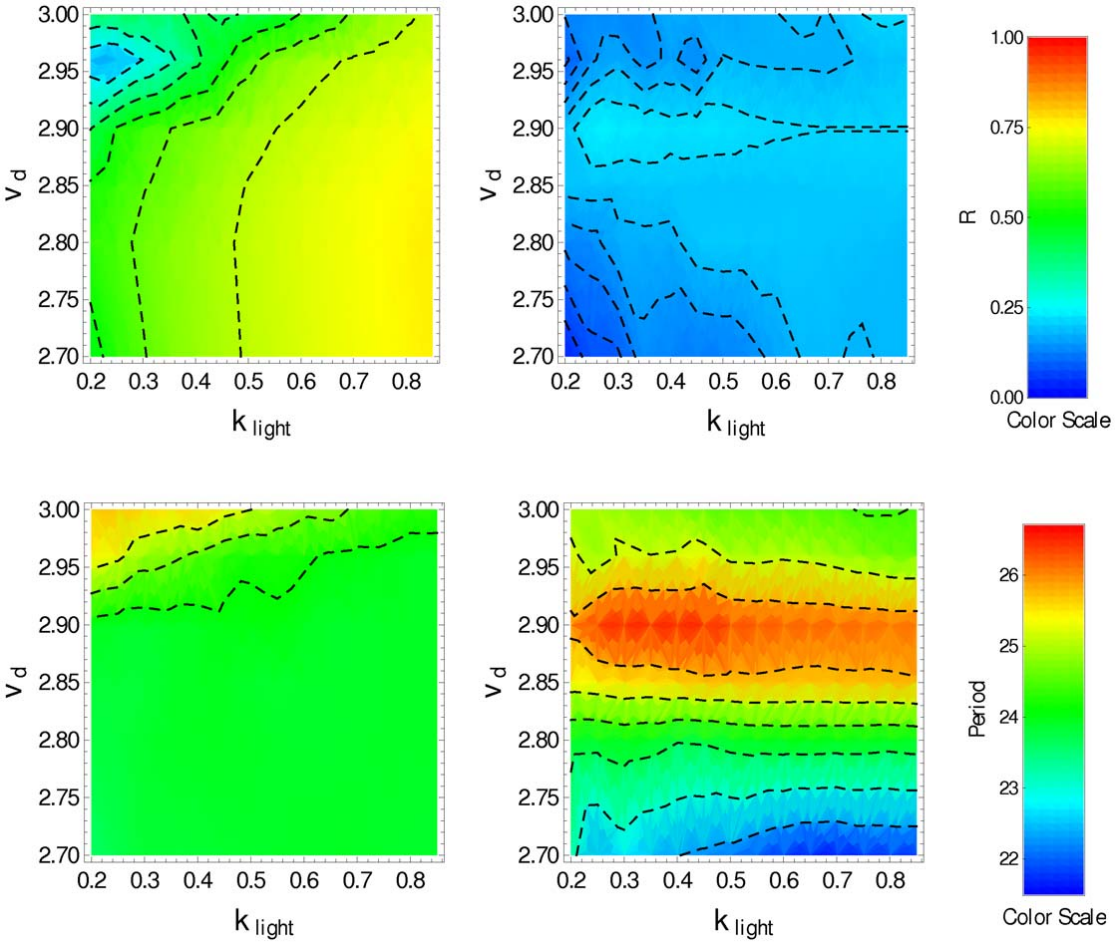
Synchronization degree and period for interacting clock neurons with 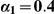
 **.** 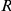 (top) and period (bottom) in the 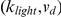-space, obtained by averaging over the cell population in the last 5 days when the transport into the nucleus, 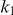, is replaced by 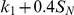. Panels on the left correspond to LD condition, and panels on the right correspond to DD condition (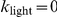).

**Figure 8:**
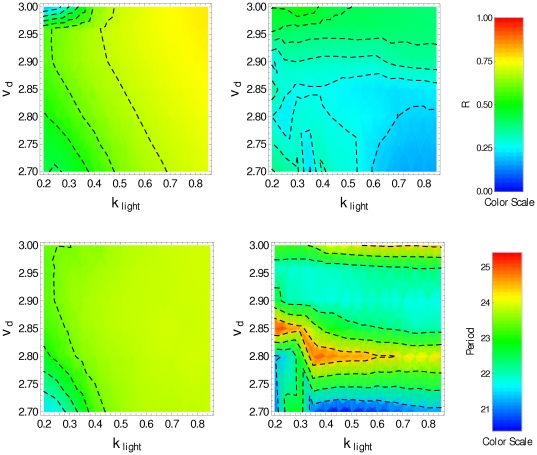
Synchronization degree and period for interacting clock neurons with 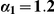
 **.** 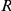 (top) and period (bottom) in the 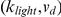-space, obtained by averaging over the cell population in the last 5 days, when the transport into the nucleus, 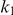, is replaced by 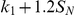. Panels on the left correspond to LD condition, and panels on the right correspond to DD condition (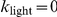).

We have also done numerical simulations for two other values of 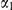: 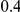 and 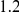. For 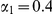, we found that under DD condition there exists circadian oscillation for 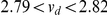, although the degree of synchronization is poor, 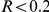 (see Fig. 7). For 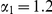 (see Fig. 8) we found that, for DD condition, there exists circadian oscillation for 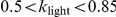 and 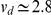 or 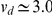, the degree of synchronization being 0.2 and 0.4, respectively. However, in these parameter regions the oscillation has a period 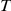 smaller than 24 h under the LD condition.

**Figure 9:**
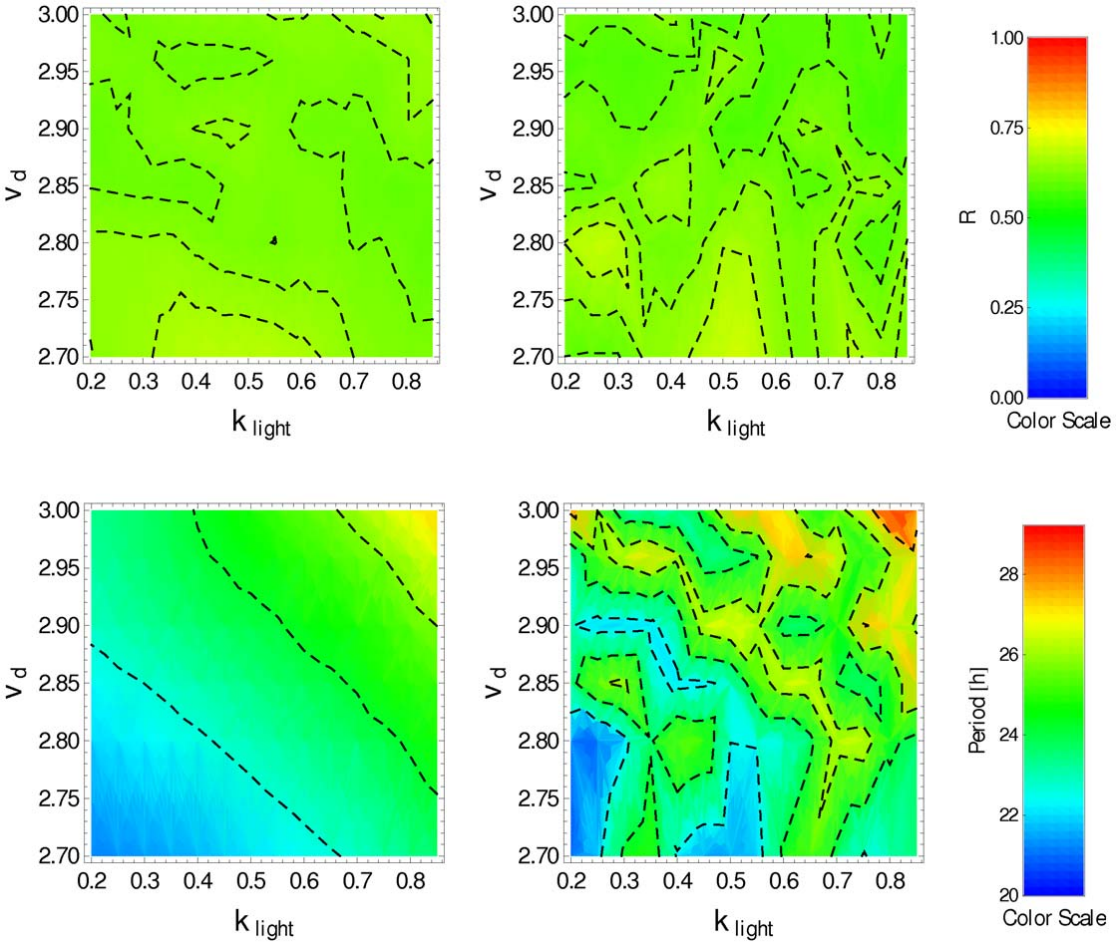
Synchronization degree and period for interacting clock neurons with 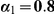
 **and** 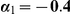 **.** 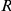 (top) and period (bottom) in the 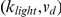-space, obtained by averaging over the cell population in the last 5 days, when the transport into the nucleus, 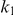, is replaced by 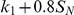, and the transport from the nucleus, 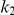, is replaced by 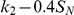. Panels on the left correspond to LD condition, and panels on the right correspond to DD condition (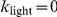).

We have also analyzed the effect of increasing 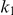 and decreasing 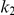 at the same time. Numerical simulations using 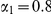 and 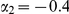 show that, for both LD and DD conditions, the degree of synchronization 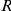 is high in the 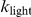 interval 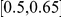 in the whole range of the parameter 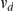 used in the simulations (top panels of Fig. 9). Circadian oscillations are obtained for both LD and DD conditions in the region around the segment that links the points (0.4,2.95) and (0.65,2.80) in the 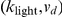-space (bottom panels of Fig. 9). The top panels of Fig. 9 suggest that this type of modulation produces better synchronization, and that the parameter regions where the system presents circadian oscillations are larger than in the case 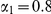 displayed in Fig. 4 for which 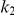 is not modulated by the input network feedback 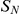.

**Figure 10:**
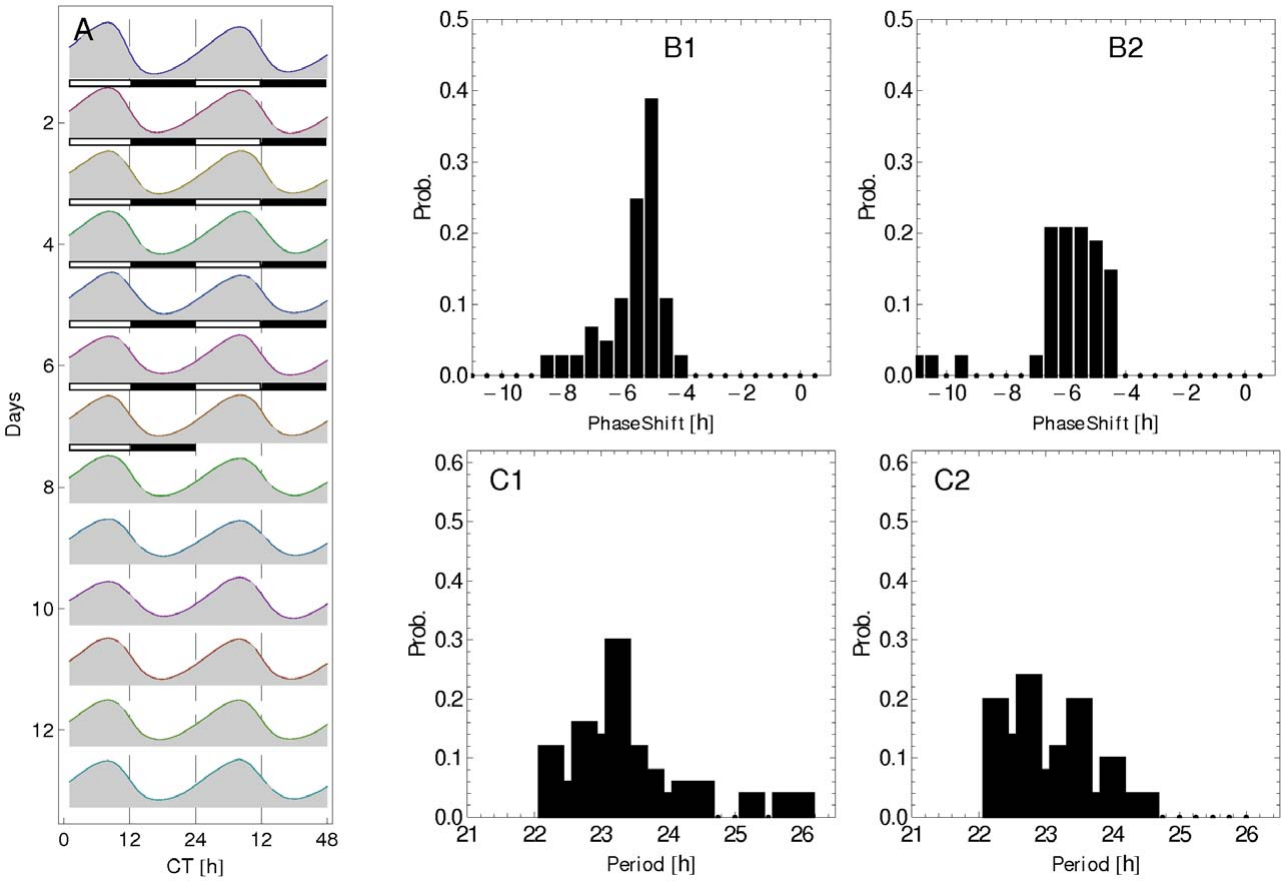
Synchronization for interacting clock neurons: Actogram and histograms. A: Double plot actogram (for 7 days under LD condition followed by 7 days under DD condition) of averaged 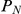 corresponding to Fig. 9 for 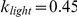, 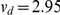 (other parameter values are given in Table 1). The 7 days under LD condition are indicated by the white-black bars. B1, B2: Histograms of phase shift. C1, C2: period obtained by fitting Eq. (2) to the time course of 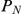 concentration at each clock neuron. B1, C1 correspond to LD condition, and B2, C2 to DD condition.

Figure 10A depicts the time course (double plot actogram) of 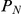 averaged over the clock neuron population when 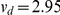 and 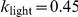. We can observe that the interneuron communication, mediated by the simultaneous modulation of 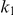 and 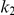, produces oscillations that preserves the period and amplitude, but introduces a phase shift of 6 h in advance. Fig. 10B shows the histogram of the phase shift, and Fig. 10C the periods of the cells. The period and phase of each clock neuron were obtained by fitting the time course of 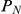 to the function (2).

**Figure 11:**
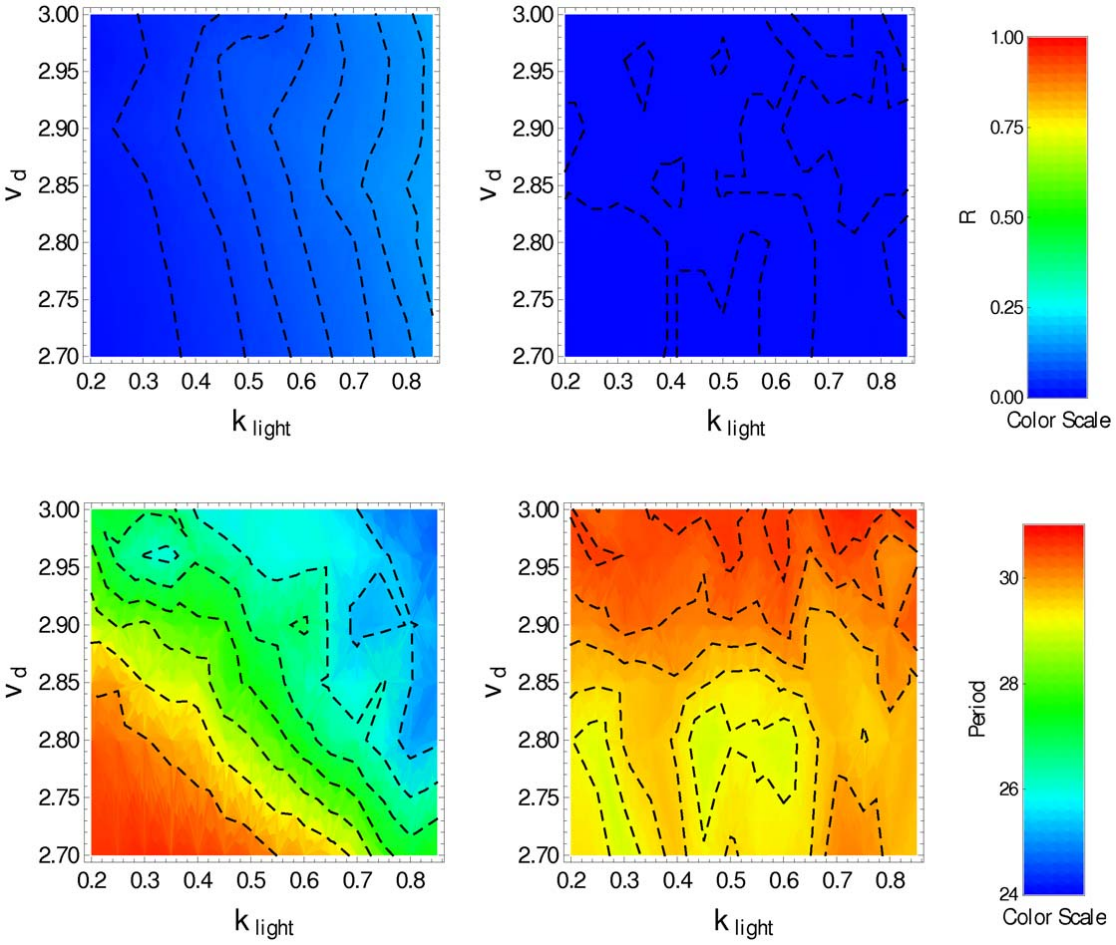
Positive modulation of non-photic degradation does not lead to synchronization. 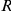
 (top) and period (bottom), in the 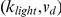-space, obtained by averaging over the cell population in the last 5 days, when the non-photic degradation 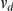 is replaced by 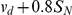. Panels on the left correspond to LD condition, and panels on the right correspond to DD condition (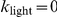).

**Figure 12:**
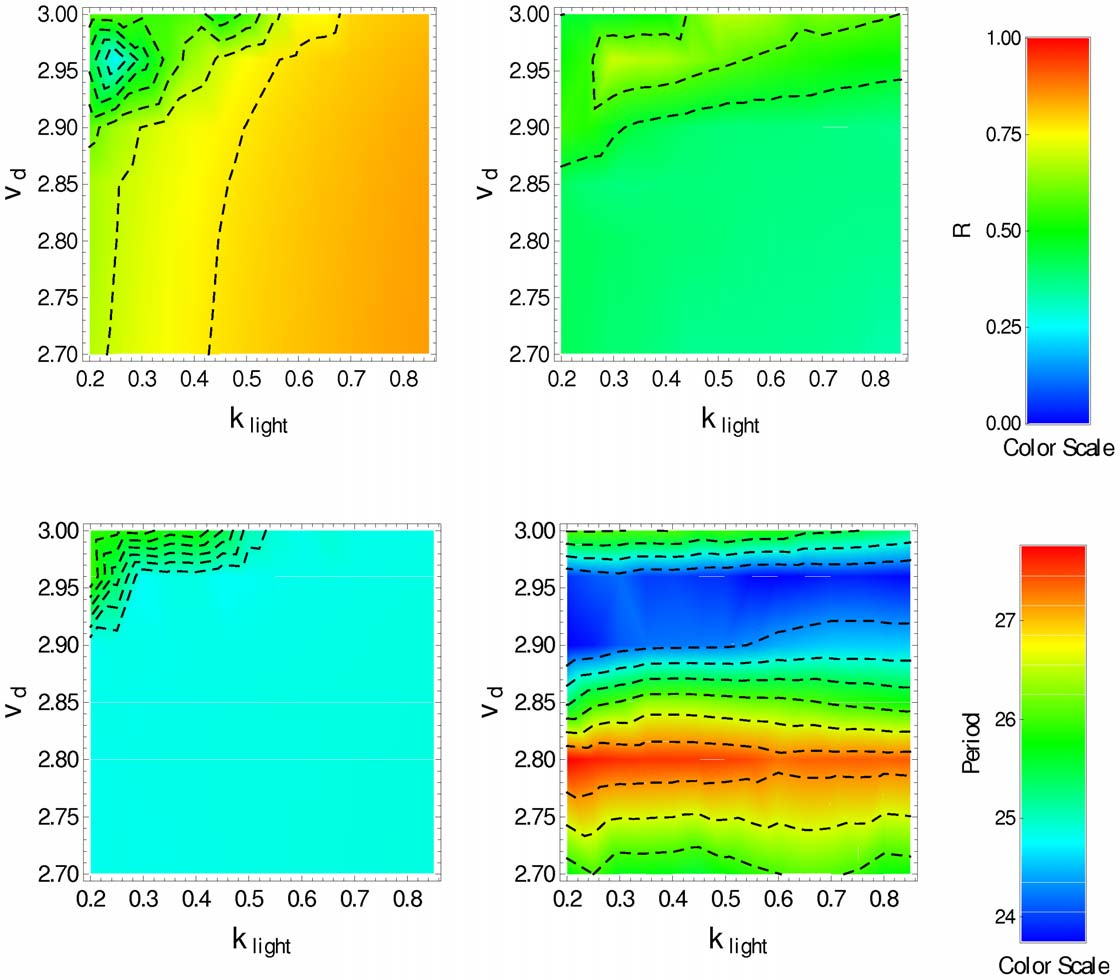
Negative modulation of non-photic degradation does not lead to synchronization. 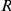
 (top) and period (bottom), in the 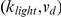-space, obtained by averaging over the cell population in the last 5 days, when the non-photic degradation rate, 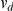, is replaced by 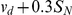. Panels on the left correspond to LD condition, and panels on the right correspond to DD condition (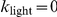).

- Now we assume that the input network feedback 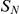 increases the non-photic degradation rate 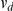, this being accomplished by replacing 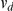 by 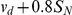 in Eqs. (1). In this case the positive modulation of the 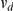 parameter does not lead to synchronization in the whole region of parameters 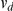 and 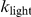 used in the simulation (Fig. 11). Furthermore, the oscillation period 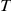 is substantially larger than 24 h. However, a decrease in the non-photic degradation 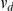 can lead to synchronization. Figure 12 displays the results obtained when 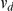 is decreased according to 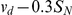, showing that in free running there is synchronization and circadian oscillation. However, for the set of parameter values studied here the associated oscillations have periods longer than 25 h.

## Discussion

During the past years many important studies have elucidated the mechanisms underlying circadian oscillations at the molecular level. These mechanisms are common to several types of cells. However, the persistence of the oscillation at tissue level is tissue specific, even though circadian rhythms at the single cell level persist for weeks in constant darkness. It is known that in the ventral lateral neuron group the neuropeptide PDF is required to maintain the circadian rhythm of this group under DD condition. However, it is not known how this neuropeptide acts to promote the synchronization of the clock neurons. In order to uncover the putative mechanisms (or discard wrong hypotheses) that allow us to explain the observed behavior in *Drosophila*, we have developed a mathematical model for a network of interacting clock neurons, where some parameters of each clock neuron are modulated by the PER protein level averaged over the whole network. Unlike other clock network models [25], which use a heterogenous population of neurons, the network in our model is formed by identical cells. The dispersion in the period and phase of the oscillations arises from the molecular fluctuations rather than from clocks with different parameters.

In contrast with [29], [30], our simple model has not considered a neurotransmitter hypothesis. The literature is scarce regarding the mechanistic details connecting the clock neuron state to the release of neurotransmitters. So our model directly uses the double-phosphorylated PER as synchronizer agent. This assumption would not adequately represent the real world. Nevertheless, it admits a more realistic interpretation: a particular state of the clock (the high concentration of PER in our simple model) promotes PDF releases.

With our model we have studied the degree of synchronization of oscillators, and the period and phase of the oscillation, at the network level, in the space of parameters for some alternative hypotheses. In particular, we have investigated the effect on the synchronization when (i) 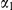 is varied so as to modulate the parameter 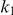 that controls the net entrance of PER into the nucleus; and (ii) 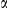 is varied so as to modulate the parameter 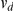 that controls the non-photic degradation of PER.

Our results indicate that for 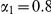, the modulation of PER entrance into the nucleus allows the synchronization of clock neurons leading to coherent circadian oscillations under constant darkness condition. These results were obtained for a network with 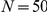, a number sensibly greater than the actual number of lateral neurons in the fly brain. However, similar circadian oscillations have been also observed for networks with a different number of clock neurons (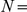
10, 25, 80, 150 and 200, data not shown). This lack of dependence on 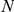 is likely due to the fact that 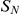 is normalized by 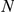. We have also tested other values of the parameter 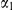: for lower modulation (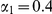) there is a poor synchronization of the clock neurons in free running, and for higher modulation (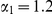) the synchronization increases but the oscillations have period shorter than 24 h. In contrast, the modulation of the non-photic degradation cannot reset the phases of individual clocks subjected to intrinsic biochemical noise.

The present network model is able to display circadian oscillation even in the absence of external *zeitgebers* when the input feedback signal 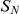 (defined as the average of 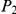 over the entire network) increases the 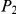 entrance rate into the nucleus. There exists a small region in the parameter space that supports circadian oscillation both under the LD and DD conditions simultaneously, the phase shift between these conditions being small. We have also observed that there exist several ways to reach a high degree of neuronal synchronization, but at the expense of noncircadian oscillations.

Our results indicate that mechanisms based on a positive feedback acting over the rate of entrance of the phosphorylated PER into the nucleus could be essential for maintaining the circadian oscillation under free-running condition. This fact suggests a putative way of action for the neuropeptide PDF, which could be acting as an agent that promotes the entrance of the phosphorylated PER into the nucleus.
